# Identification, Evolutionary Dynamics, and Gene Expression Patterns of the *ACP* Gene Family in Responding to Salt Stress in *Brassica* Genus

**DOI:** 10.3390/plants13070950

**Published:** 2024-03-25

**Authors:** Fang Qian, Dan Zuo, Tuo Zeng, Lei Gu, Hongcheng Wang, Xuye Du, Bin Zhu, Jing Ou

**Affiliations:** 1 School of Life Sciences, Guizhou Normal University, Guiyang 550025, China; 82101221107@caas.cn (F.Q.); 21010100449@gznu.edu.cn (D.Z.); zengtuo@gznu.edu.cn (T.Z.); leigu1216@nwafu.edu.cn (L.G.); duxuye@gznu.edu.cn (X.D.); 201703008@gznu.edu.cn (B.Z.); 2 College of Forestry, Guizhou University, Guiyang 550025, China

**Keywords:** acyl carrier proteins, *Brassica*, gene family, *Brassica napus*, gene expression

## Abstract

Acyl carrier proteins (ACPs) have been reported to play a crucial role in responding to biotic and abiotic stresses, regulating growth and development. However, the biological function of the *ACP* gene family in the *Brassica* genus has been limited until now. In this study, we conducted a comprehensive analysis and identified a total of 120 *ACP* genes across six species in the *Brassica* genus. Among these, there were 27, 26, and 30 *ACP* genes in the allotetraploid *B. napus*, *B. juncea*, and *B. carinata*, respectively, and 14, 13, and 10 *ACP* genes in the diploid *B. rapa*, *B. oleracea*, and *B. nigra*, respectively. These *ACP* genes were further classified into six subclades, each containing conserved motifs and domains. Interestingly, the majority of *ACP* genes exhibited high conservation among the six species, suggesting that the genome evolution and polyploidization processes had relatively minor effects on the *ACP* gene family. The duplication modes of the six *Brassica* species were diverse, and the expansion of most *ACPs* in *Brassica* occurred primarily through dispersed duplication (DSD) events. Furthermore, most of the *ACP* genes were under purifying selection during the process of evolution. Subcellular localization experiments demonstrated that *ACP* genes in *Brassica* species are localized in chloroplasts and mitochondria. *Cis*-acting element analysis revealed that most of the *ACP* genes were associated with various abiotic stresses. Additionally, RNA-seq data revealed differential expression levels of *BnaACP* genes across various tissues in *B. napus*, with particularly high expression in seeds and buds. qRT-PCR analysis further indicated that *BnaACP* genes play a significant role in salt stress tolerance. These findings provide a comprehensive understanding of *ACP* genes in *Brassica* plants and will facilitate further functional analysis of these genes.

## 1. Introduction

Acyl carrier proteins (ACPs) are acidic proteins found in organisms, and they play important roles in regulating growth and development, as well as in responding to biotic and abiotic stresses [1,2]. ACPs have a tertiary structure comprising a short transverse α-helix and three parallel α-helices. This structure creates a hydrophobic cavity and allows for significant structural adaptability. Consequently, ACPs can accommodate acyl chains of varying lengths, bind to acyl intermediates, and transfer between the active sites of different enzymes [3,4].

Studies have shown that ACPs play a crucial role in fatty acid synthesis by carrying acyl chains and facilitating enzymatic reactions. ACP acts as a cofactor in acyl ACP desaturation reactions and performs plastid acyl transferase-like actions with fatty acids of varying acyl chain lengths [5]. Moreover, ACPs are essential proteins for the cycle of fatty acid chain elongation and reduction. They effectively bind to the acyl chain and transport it to the active sites of various enzymes [6]. In a study where the gene expression of *ACP4* was inhibited using RNAi, there was a decrease in the leaf fat content of *Arabidopsis* (*Arabidopsis thaliana*) and a decrease in the proportion of 16:3 in the total fatty acid composition [7]. Fibre-specific *ACP* in cotton (*Gossypium hirsutum*) positively regulates cotton fibre elongation by improving the synthesis of membrane lipids [8]. In the pepper plant (*Capsicum chinense*), the *ACP* gene plays a crucial role in the production of branched fatty acid chains, which are necessary for the synthesis of capsaicin [9]. Additionally, a study conducted on *Brassica napus* revealed that multiple ACPs can fulfil the requirements for fatty acid synthesis [10]. Recent research has also highlighted the essential role of *CsACP1* (ACP1 in *Coriandrum sativum*) in the synthesis of distinct monoenoic fatty acids found in seed oil [11].

Recently, several studies have revealed that ACP also plays crucial roles in enhancing plant tolerance to various stresses [12,13,14,15]. The *ACP* genes that exhibit tolerance to abiotic stresses have been extensively studied in *Arabidopsis*. Notably, *AtACP1*, *AtACP2*, and *AtACP3* have shown high gene expression levels in response to drought, indicating that these genes are involved in drought tolerance. In the presence of salt stress, the *atacp5* mutant displays increased sensitivity compared to that of the wild type, while overexpression of *AtACP5* enhances salt stress tolerance in transgenic *Arabidopsis*. Moreover, recent studies have identified certain *SbACP* (ACP in *Sorghum bicolor*) genes that contribute to drought and salt stress tolerance [16].

The *Brassica* genus is highly valued for its economically important species, including those used for food, oil, vegetables, ornamentals, and fodder [17,18]. Within this genus, there are six closely related species that are particularly significant and can be represented by the U triangle [19]. The U triangle consists of three fundamental diploid species: *B. rapa* (AA, 2n = 2x = 20), *B. nigra* (BB, 2n = 2x = 16), and *B. oleracea* (CC, 2n = 2x = 18). Additionally, there are three amphidiploids: *B. napus* (AACC, 2n = 4x = 38), *B. juncea* (AABB, 2n = 4x = 36), and *B. carinata* (BBCC, 2n = 4x = 34). These amphidiploids were created through hybridization between any two of the three diploid species. Moreover, these amphidiploids can be resynthesized using interspecific crosses involving diploid species, which makes them an excellent and widely used model for allopolyploid analysis. All of the genomes of the species in the triangle of U model have been well sequenced [20,21,22,23,24,25] and serve as valuable resources for studies involving gene function and genome evolution in *Brassica*. At the same time, it is also convenient for us to study the biological function of the *ACP* genes. At present, the function of *ACP* genes in the fatty acid synthesis pathway of *Brassica* species has been less explained, but the potential function in response to abiotic stress has not been investigated. Although the *ACP* gene family has been extensively studied in numerous species, the evolutionary history and gene functions of *ACP* genes in the six cultivated *Brassica* species have not been investigated thoroughly. The accessibility of multigenome sequencing allows us to identify potential *ACP* orthologous and paralogous genes. Additionally, RNA-seq data facilitate the examination of functional correlations among *ACP* genes. In this study, we systematically identified and analyzed the *ACP* gene family across the six cultivated species in the triangle of U. Furthermore, we conducted a comprehensive analysis of *ACP* expression patterns in *B. napus* under salt stress, which provides a solid foundation for further investigation into the molecular mechanisms underlying *ACP* responses to salt stress.

## 2. Results

### 2.1. Identification and Characteristics of ACP Genes in the Brassica Genus

To identify putative *ACP* genes in six cultivated *Brassica* species, we used ACP protein sequences from *Arabidopsis* and the PP-binding domain of ACP proteins (PF00550) as queries to search for ACP proteins across the genomes of the *Brassica* species. In total, 120 *ACP* genes were identified from six *Brassica* species (Table S1), including 14, 10, and 13 genes in the diploid progenitors *B. rapa*, *B. nigra*, and *B. oleracea* and 26, 27, and 30 *ACP* genes in the allotetraploid *B. juncea*, *B. napus*, and *B. carinata*. These identified *ACP* genes in six *Brassica* species were uniformly named according to their homologous relationship with the *ACP* genes of *Arabidopsis* and their origin from different *Brassica* species. For example, “*BcamtACP2.1*” indicates that this gene from *B. carinata* is highly homologous to *mtACP2* in *Arabidopsis*.

The physicochemical properties of ACP family proteins in *Brassica* were analyzed. This analysis included the molecular weight (MW), theoretical isoelectric point (pI), instability coefficient (II), and protein average hydrophobicity index (GRAVY) (Table S1). The MW of these ACP proteins ranged from 12.93 kDa (BniACP2) to 21.57 kDa (BcamtACP3.2), while the theoretical isoelectric points ranged from 4.42 (BnaACP4.3) to 7.09 (BjumtACP2.3). Except for BjumtACP2.3, most ACP proteins were acidic (pI < 7), indicating that the ACP proteins in *Brassica* species had low molecular weight acidic proteins. Approximately 25.83% of the ACP proteins in *Brassica* were classified as stable proteins (instability index < 40), with the protein instability coefficient ranging from 29.41 to 61.86. The average hydrophobicity index of these ACP proteins ranged from −0.38 to 0.124, with the majority (96.67%) exhibiting weak hydrophilicity.

### 2.2. Subcellular Localization Analysis of ACP Family Proteins in B. napus

**Figure 1:**
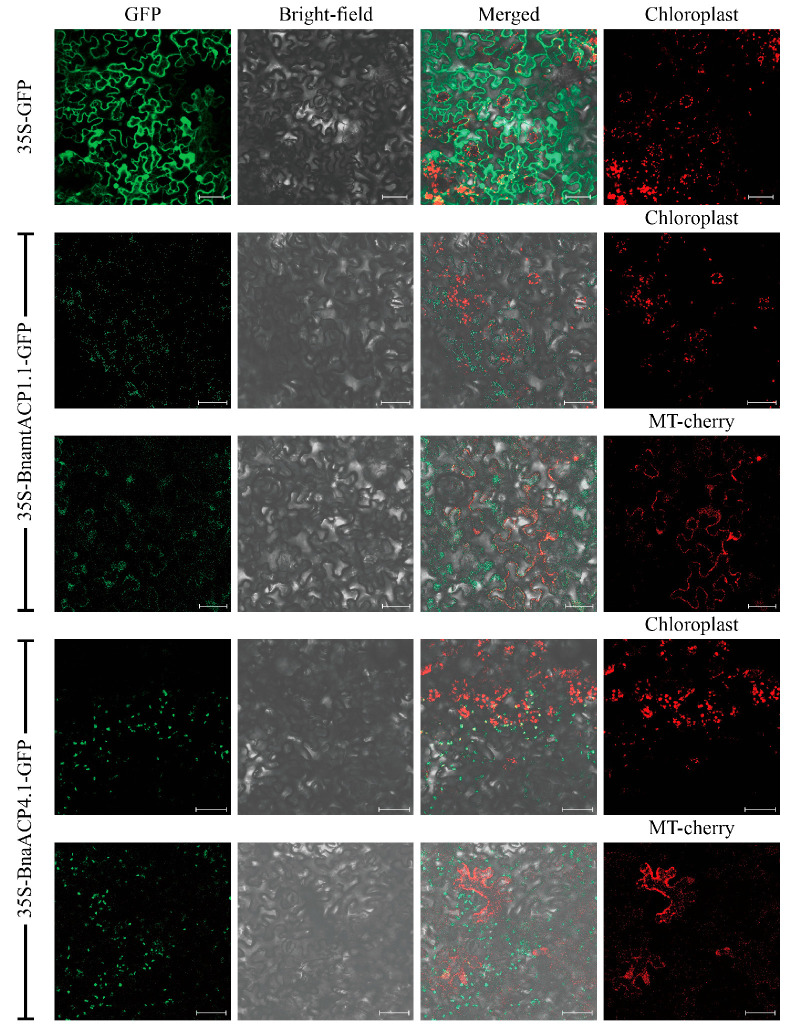
Subcellular localization of two BnaACP proteins in tobacco. Chloroplast: chloroplast auto-fluorescence; MT-mcherry: mitochondria-specific marker-labelled recombinant plasmid. Scale bar = 50 µm.

The subcellular localization of ACP proteins in *Brassica* plants was investigated, revealing that 22 ACP proteins were located in mitochondria, while 98 ACP proteins were localized in chloroplasts (Table S1). To confirm their subcellular localization, six BnaACPs tagged with green fluorescent protein (GFP) (BnaACP-GFP) were constructed. These constructs, along with a recombinant plasmid labelled with a mitochondria-specific marker, were used to infect the leaf epidermal cells of tobacco. Confocal microscopy was used to determine the expressed localizations of the BnaACPs. The results showed that BnaACP3.1, BnaACP4.1, and BnaACP5.1 GFP signals were observed only in combination with chloroplast autofluorescence signals. On the other hand, BnamtACP1.1, BnamtACP2.1, and BnamtACP3.1 GFP signals were observed only in combination with mitochondrial-specific marker signals. These findings indicate that the selected BnaACPs were located in both chloroplasts and mitochondria (Figure 1 and Figure S1).

### 2.3. Phylogenetic Analysis of ACP Proteins

**Figure 2:**
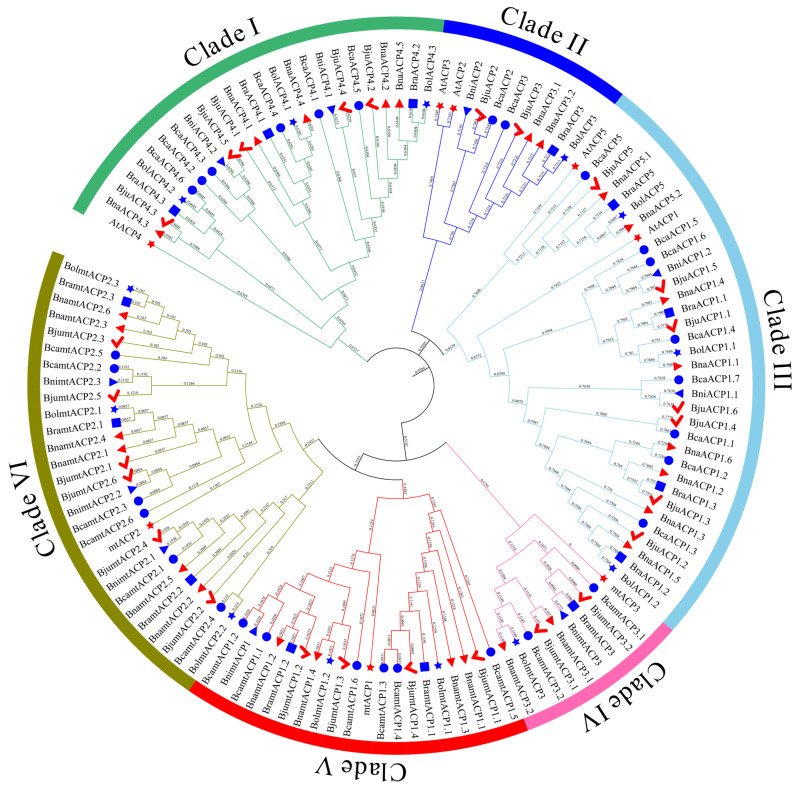
Phylogenetic tree of ACP family proteins from *Arabidopsis* and six *Brassica* species. Red star, blue circle, red checkmark, red right pointing triangle, blue left pointing triangle, blue star, and blue rectangle represent ACP family proteins from *Arabidopsis*, *B. carinata*, *B. juncea*, *B. napus*, *B. nigra*, *B. oleracea*, and *B. rapa*, respectively.

To investigate the evolutionary relationship of ACP proteins in *Brassica*, a phylogenetic tree was constructed using the maximum likelihood (ML) method. The ML tree included 120 ACP proteins from six *Brassica* species and eight ACP proteins from *Arabidopsis*. The analysis revealed that all the ACP proteins in *Brassica* and *Arabidopsis* could be categorized into six subclades (Clade I - Clade VI). Most orthologues were found to be clustered together in the same subclade (Figure 2). Clade I consisted solely of ACP4 orthologous proteins, with a total of 25 ACP proteins. Clade II contained the ACP3 (7 ACP proteins) and ACP2 (4 ACP proteins) orthologous proteins. Clade III had the highest number of ACP family protein members, including the 3 BolACP, 2 AtACP, 2 BniACP, 4 BraACP, 7 BjuACP, 8 BcaACP, and 8 BnaACP proteins. Furthermore, clades IV, V, and VI consisted of the 10 mtACP3, 20 mtACP1, and 28 mtACP2 orthologous proteins, respectively.

### 2.4. Conserved Motif, Conserved Domain, and Gene Structure Analysis of ACP Family Proteins

**Figure 3:**
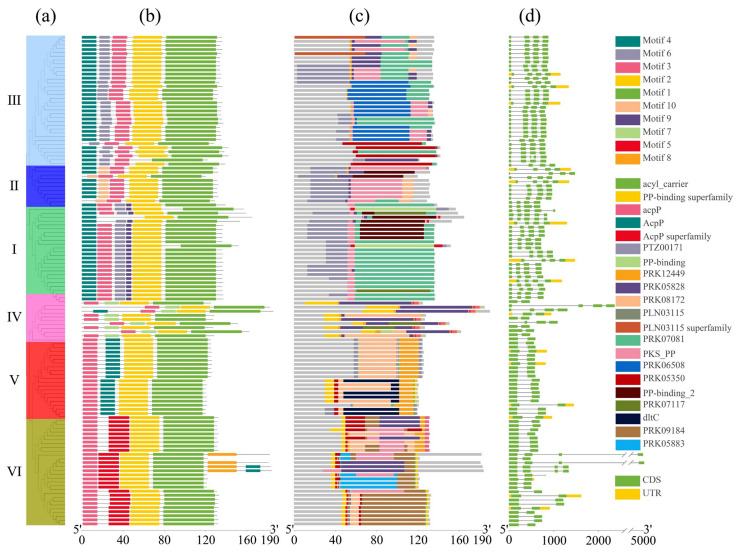
Detailed structure of ACP family proteins in six cultivated species of *Brassica*. (**a**) Phylogenetic tree, (**b**) conserved motif, (**c**) conserved domain, (**d**) gene structure.

The conserved motifs within the ACP family proteins in *Brassica* were analyzed, and 10 conserved amino acid sequence regions (motifs) were identified across these ACP proteins (Figure 3a,b). The detailed amino acid sequence of these motifs is listed in Figure S2. Notably, we observed a higher genetic relationship corresponded to a greater similarity in motif structure. Family members within the same clade exhibited higher similarity, indicating that the structure of motifs in orthologous ACP proteins was more conserved than that in paralogous ACP proteins. Motif 4, motif 6, motif 3, motif 2, and motif 1 were present in all of these ACP proteins clustered in clade III, except in BcaACP5 (missing motif 4) and in BnaACP5.2 (missing motif 2). Motifs 10, 9, 7, and 5 were specific to the proteins clustered in clade II, clade I, clade IV, and clade VI, respectively. Moreover, motif 8 is an infrequent motif that was detected only in four ACP proteins (BolmtACP2.3, BnamtACP2.6, BnamtACP2.3, and BjumtACP2.3). The ACP proteins in clade IV showed an unstable arrangement and different motifs compared to the ACP proteins in the other clades. Conserved domain analysis (Figure 3c) revealed that the PTZ00171 domain was present in all the ACP proteins, indicating its specific structure for ACP family proteins. In addition, we also investigated the structural diversity of the identified ACP genes and analyzed their exon–intron structure, which demonstrated significant variation in the number of introns among the ACP families in *Brassica* compared to that among their orthologues in *Arabidopsis* (Figure 3d). Moreover, the similarity in gene structure among the *ACP* genes is positively correlated with their genetic relationship.

### 2.5. Chromosomal Localization Analysis of ACP Genes

**Figure 4:**
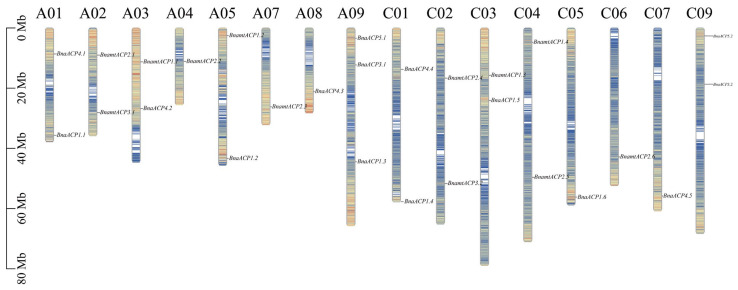
Chromosome distribution of *ACP* genes in *B. napus*. Chromosomes without *ACP* gene distribution (A06, A10, and C08) are not shown. The different colours on the chromosomes represent the gene density.

To visualize the location of *ACP* genes in chromosomes, the physical location of these genes was determined based on the *Brassica* genome annotation (gff) files. Out of 120 *ACP* genes, 119 were successfully mapped to assembled chromosomes (Figure 4 and Figure S3 and Table S1). However, *BjuACP1.1* was found on unassembled scaffolds. We observed that the majority of the *ACP* genes and their locations in amphidiploid *B. napus* and *B. juncea* corresponded to those in the ancestral diploid genome, but the gene position and gene number were relatively unstable in *B. carinata*, in which several *ACP* genes were lost in the C subgenome and some *ACP* genes were duplicated in the B subgenome when compared to their orthologues in the diploid genomes. In addition, most *ACP* genes were located in regions with a high density of genes.

### 2.6. Cis-Acting Elements of ACP Genes

**Figure 5:**
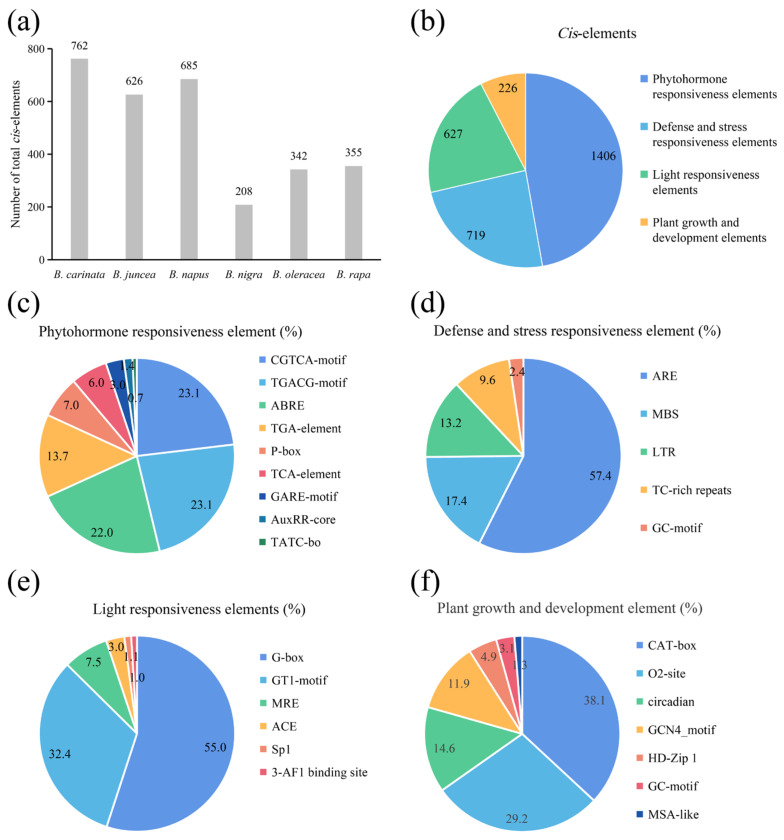
The prediction results of the *cis*-acting elements of the *ACP* gene family in *Brassica*. (**a**) Promoter elements of all *ACP* genes in each species. (**b**) Classification of all promoter elements in six species. (**c**–**f**) Details of all promoter classifications.

To detect the potential molecular functions of the *ACP* genes in *Brassica*, the 1.5 kb promoter sequence upstream of each *ACP* was extracted, and the distributions of *cis*-acting elements were analyzed. The results showed that a total of 2987 *cis*-acting elements ranging from 208 (*B. nigra*) to 762 (*B. carinata*) in the *ACP* genes were identified in these *Brassica* species (Figure 5a). These elements were primarily associated with hormone regulation (1406 elements of 2987 in total), stress resistance (719), light responsiveness (627), and growth and development (226) in sequence (Figure 5b). Within the hormone regulation group (Figure 5c), the CGTCA-motif (23.1%), TGACG-motif (23.1%), and ABRE (22.0%) were the most overrepresented elements. In the stress resistance group (Figure 5d), these elements were predominantly represented by AREs (57.4%), followed by MBSs (17.4%). In the light responsiveness group (Figure 5e), the G-box (55.0%) and GT1-motif (32.4%) were the dominant elements. In the plant growth and development group (Figure 5f), these elements were overrepresented by CAT-box (38.1%), O2-site (29.2%), and circadian (14.6%). The presence of multiple *cis*-acting elements indicates that the *ACP* genes in *Brassica* plants serve multiple functions, particularly the function of stress responsiveness.

### 2.7. Gene Duplication, Synteny, and Evolution Analysis of ACP Genes in Six Brassica Species

Angiosperms have been demonstrated to undergo multiple rounds of whole-genome duplication, resulting in the expansion of gene families. To investigate the replication events of *ACP* genes in *Brassica* species, we employed DupGen_finder [26] to analyze the occurrence of tandem duplication (TD), whole-genome duplication (WGD), proximal duplication (PD), transposed duplication (TRD), and dispersed duplication (DSD) genes in the ACP family of six *Brassica* species (Table S2). We did not find any TD, WGD, PD, or TRD events in the six *Brassica* species, but DSD events were observed in all of them. Among the DSD events in the *ACP* gene family across the six species, the highest proportion was 26.42% (28/106) in *B. carinata*, while the lowest proportion was 7.55% (8/106) in *B. nigra* compared to the other five species. This finding suggests that in the genomes of the six *Brassica* species, the *ACP* genes primarily contribute to the expansion of the gene family through DSD events.

**Figure 6:**
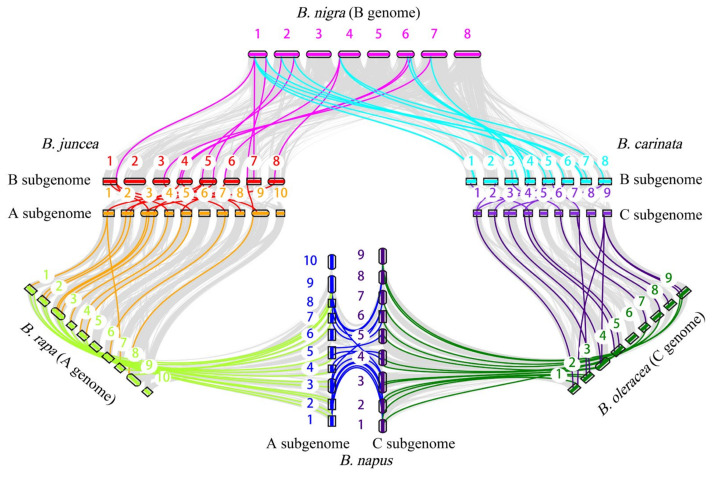
Collinearity analysis of *ACP* genes in six *Brassica* species.

To identify the intraspecific and interspecific collinear gene pairs between three diploid species and their corresponding tetraploid species, we compared the *ACP* genes of the diploid species with those of the three allotetraploid species. We also identified collinear gene pairs within each species. The collinear gene pairs of *ACP* genes were then visualized (Figure 6). The intraspecific collinear gene pairs included 7 gene pairs in *B. rapa*, 4 in *B. oleracea*, 2 in *B. nigra*, 17 in *B. napus*, 18 in *B. juncea*, and 21 in *B. carinata* (Table S3). In addition, 13 pairs of syntenic orthologous genes were found in *B. nigra* and *B. oleracea*, but only 11 pairs were found in the two subgenomes of *B. carinata*, suggesting that some of the collinear *ACP* genes might have been lost or that homologous exchange (HEs) might have occurred during the process of polyploidization.

**Figure 7:**
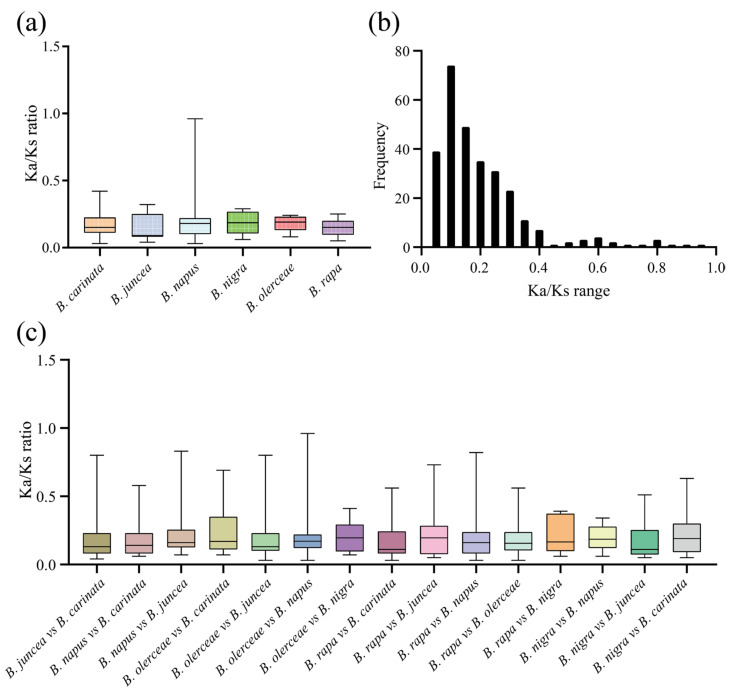
The Ka/Ks values of *ACP* genes in six *Brassica* species. (**a**) Analysis of intraspecific Ka/Ks ratios. (**b**) Frequency distribution of the Ka/Ks values. (**c**) Analysis of interspecies Ka/Ks ratios.

The nonsynonymous (Ka) and synonymous (Ks) nucleotide substitution patterns of protein-coding genes are important indicators of gene evolution. The Ka/Ks ratio is used in genetics to determine whether there are selective pressures on protein-coding genes and evaluate the gene divergence rates. A Ka/Ks ratio > 1 indicates positive selection, a Ka/Ks ratio = 1 indicates neutral selection, and a Ka/Ks < 1 indicates purifying selection [27]. In this study, we calculated the Ka/Ks values for duplicated gene pairs to investigate whether the *ACP* genes were associated with selective pressure after duplication events. First, we obtained the intraspecies Ka/Ks values in six *Brassica ACP* families (Figure 7a; Table S4). After removing genes with a Ka or Ks of 0, the average Ka/Ks ratio was 0.17. The Ka/Ks values of the ACP genes among the different species ranged from 0.03 to 0.96, with the majority falling within the range of 0.05–0.3 (Figure 7b). Next, we calculated the interspecies Ka/Ks values in the six *Brassica* ACP families (Figure 7c; Table S5). Similarly, genes with a Ka or Ks of 0 were removed. These results implied that these *ACP* genes were subject to strong purifying selection pressures, both intraspecies and interspecies, in the six *Brassica* species.

### 2.8. Tissue-Specific Expression of ACP Genes

The *B. napus* multi-omics information resource (BnIR) database provides a comprehensive knowledge base of expression data for *BnaACP* (*ACP* genes in *B. napus*) genes from various tissues, including rosette, roots, stems, leaves, cotyledons, buds, sepals, petals, filaments, pollen, siliques, and seeds. This database allows for the detection of tissue expression patterns of *ACP* genes. The expression heatmap (Figure S4) revealed distinct temporal and spatial expression characteristics of *BnaACP* genes among different tissues. In general, two-thirds of *mtACPs* exhibited relatively low expression in all of the tested tissues. Notably, all of the *BnaACP4* paralogues except *BnaACP4.3* showed high expression levels in cotyledons and vegetative rosettes, suggesting that *ACP4* genes actively function in vegetative growth. *BnaACP4* genes are primarily expressed in vegetative tissues, and *BnaACP4.3* is also enriched in vegetative tissues. However, *BnaACP1.1* and *BnaACP1.4* appear to express specifically in bud and seeds. Moreover, the majority of *BnaACP1* and *BnaACP3* paralogues exhibited relatively high expression in buds and seeds compared to other tissues, suggesting that these genes play a significant role in fatty acid synthesis.

### 2.9. Expression Patterns of ACP Genes in B. napus under Salts

**Figure 8:**
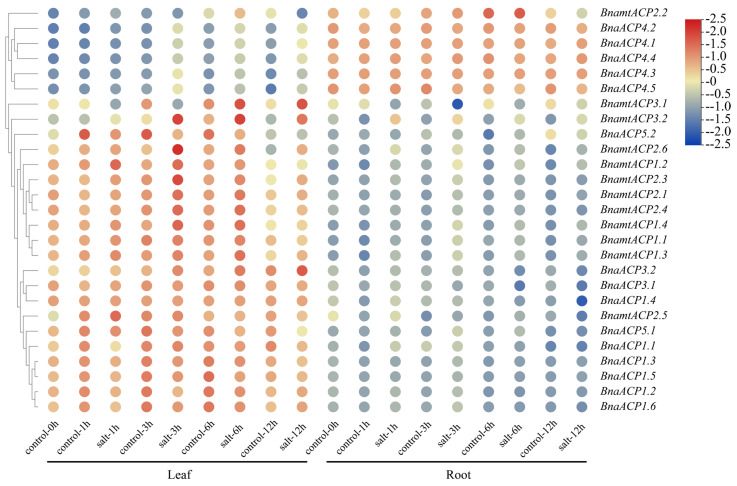
Expression patterns of the *BnaACP* genes under salt stress. The expression level of *BnaACP* genes in leaves and roots after 1 h, 3 h, 6 h, and 12 h of treatment with salt stress.

The analysis of the *cis*-elements of *ACP* genes in *Brassica* showed that *BnaACP* genes may respond to various stresses. To further detect the functions of *ACP* genes, we constructed a heatmap of *BnaACPs* expression (Figure 8) under salt stress based on the gene expression data from BnIR database. We found that approximately 80% of *BnaACPs* exhibited a relatively high expression in leaves but a low expression in roots. Moreover, almost all the *BnaACPs* genes in the leaves and roots showed different gene expression profiles, suggesting that the *ACP* genes were tissue-specific expression genes. Under salt stress, the expression level of most *BnaACPs* did not show significant changes in roots and leaves when compared to the controls. However, we observed that the expression levels of some *BnaACPs* were increased (such as *BnaACP 5.1*), while the expression levels of some *BnaACPs* were decreased (such as *BnaACP 4.1*) under salt stress, suggesting these genes are likely to be involved in responding to salt stress.

**Figure 9:**
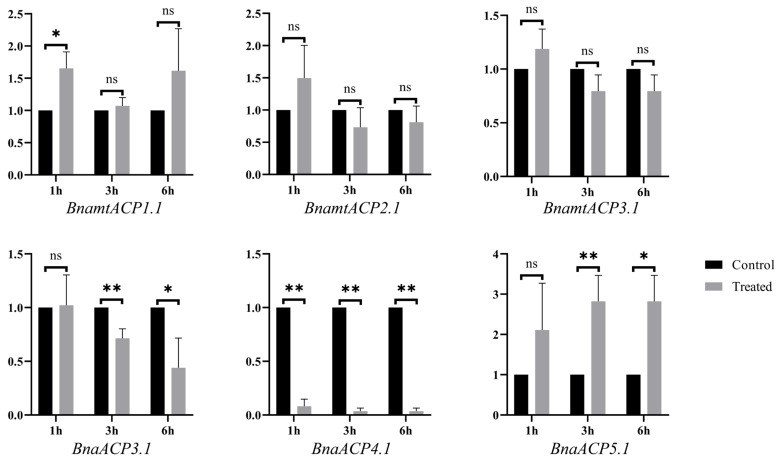
The relative expression of *BnaACP* genes under salt stress treatment. * and ** indicate significant differences at the 5% and 1% level by *t*-test. ns indicates not significant by *t*-test.

To further verify the functions of *ACP* genes, a qRT–PCR analysis of *BnaACP* genes of seedlings subjected to salt stress was conducted. According to the homology with the *ACP* genes in *A. thaliana*, we selected three *BnaACP* genes and three *BnamtACP* genes for qPCR verification (Table S6). The results (Figure 9) showed that except for *BnamtACP1.1* and *BnaACP4.1*, these selected genes did not change significantly after salt treatment for 1 h. Under treatment for 3 h and 6 h, the expression levels of both *BnaACP3.1* and *BnaACP4.1* were significantly down-regulated, while the opposite was observed for the *BnaACP5.1*. It is worth noting that *BnaACP4.1* showed a significantly decrease (*p* < 0.01) at all stress time points. Similar result for *AtACP4* was observed under iron deficiency and nitrogen starvation stresses [14]. These results demonstrate that the expression patterns of *BnaACP* genes are dynamically altered in response to salt stress. Specifically, *BnaACP4.1* may act as a negative regulator in *B. napus* salt stress response.

## 3. Discussion

The six main cultivars of the *Brassica* genus in the triangle of U are important sources of vegetable oil and emerging sources of biodiesel and biofuel for industrial production [28,29]. Fatty acids, which are the main constituents of lipid molecules, are essential nutrients for the survival of living organisms and play a crucial role in the development and growth of plants. They also provide the energy needed by biological bodies and serve as key components of cell tissues. ACP family proteins are involved in the synthesis of plant fatty acids and facilitate the transfer of acyl intermediates during the synthesis process. They play a crucial role in the direct synthesis pathway of long-chain fatty acids and also have significant involvement in other cellular metabolic processes [30,31]. To date, numerous studies have focused on identifying and analyzing ACP family proteins in various plant species [16,32,33,34], but there is a lack of research on the function and potential role of *ACP* gene families, specifically in *Brassica*. It is worth noting that the genomes of six *Brassica* species have already been sequenced and assembled [20,21,22,23,24,25]. The use of the three natural allotetraploids and their diploid progenitors in the triangle of U has been instrumental in studying scientific problems related to polyploidization. Therefore, it is crucial to conduct a comprehensive investigation to identify and analyze the *ACP* gene families in *Brassica*.

In this study, a total of 120 *ACP* genes were identified from the triangle of U model. These genes were divided into six subclades based on phylogenetic relationships. The structural diversity of the genes reflects their functional diversity and plays a significant role in the evolution of gene families. Among these subclades, each subclade contained the same conserved motifs and domains but with some degree of variation between different subclades (Figure 2). These results showed that these *ACP* genes were highly conserved in key regions but with a degree of variation in sequence structure. This indicates that the *ACP* gene families might have undergone changes in gene structure and function during evolution. Notably, the number of *ACP* family members in subclade I and subclade III of the three diploid species was approximately 1.5 times higher than that in *Arabidopsis*, while the number of *ACP* genes from the three double diploid species was approximately 4 times higher than that in *Arabidopsis*. Additionally, these findings revealed that the gene families exhibited varying numbers of exons and introns and that certain *Brassica ACP* genes lacked introns. Furthermore, RNA-Seq analysis of *B. napus* in different tissues revealed diverse and tissue-specific expression patterns of *BnaACP* genes, highlighting the functional diversity of *BnaACP*.

The *Brassica* genus shares a common ancestor with *Arabidopsis* that underwent three whole-genome duplication (WGD) events (α, β, and γ) [21]. After diverging from the *Arabidopsis* lineage, *Brassica* plants experienced a lineage-specific whole-genome triplication (WGT) event approximately 15.9 million years ago (MYA). According to the U model triangle, a typical ancestral region in *Arabidopsis* would correspond to three regions in the *Brassica* genome [21,35,36,37]. The quantitative analysis revealed that the *ACP* gene family underwent significant expansion during the process of allopolyploidy. However, the total number of *ACP* genes in *B. rapa*, *B. oleracea*, and *B. nigra* is 14, 13, and 10, respectively, which is not three times the number found in *Arabidopsis*. This indicates that the *ACP* genes experienced a loss of copy number following the whole-genome triplication. For example, the *ACP2* gene was lost during allopolyploidy in *B*. *napus*. Similar phenomena have been observed in the *MADS-box* [38] and *WRKY* [39] transcription factor families of Chinese cabbage, as well as the *MYB28* [40] family of *Brassica*. Fragment repeats and tandem duplication (TD) events have been reported to contribute to the amplification of *ZmPRXs* [41] in maize and *PbPRXs* [42] in Chinese pear (*Pyrus bretschneideri*). Our observations indicate that *ACP* genes in *Brassica* primarily undergo gene family expansion through dispersed duplication (DSD) events, suggesting that DSD events have played a major role in the expansion of the *ACP* gene family in *Brassica*. Functionally, these genes may be lost or undergo new functionalization and subfunctionalization [43]. Moreover, these new gene functions may help plants better adapt to changing natural environments, thereby preventing extinction [44].

In bacteria, ACP exists as an independent soluble protein in the type II fatty acid synthase system, while in mammals, it exists as a domain within the type I fatty acid synthase [45]. ACP is a critical member of the carrier protein family and directly participates in the synthesis of long-chain fatty acids. Plant cells possess fatty acid synthase complexes, particularly in plastids and mitochondria. The type II fatty acid synthase complex in plastids gas been extensively studied. ACP plays a crucial role in the process of fatty acid synthesis in *Brassica* crops; for example, *B. napus* could encode multiple copies (this study found 27) of *ACP* to meet the demands of fatty acid synthesis that occurred during oilseed development [10]. In transgenic *Brassica*, the prime increase was found for linolenic (C18:3) in leaves. Interestingly, transgenic approaches could be used to improve the seed oil quality of *Brassica*, mainly by increasing the ratio of monounsaturated (C18:1)/saturated fatty acids and reducing the content of erucic acid (C22:1) [46]. Overexpression of *AtACP5* also led to changes in fatty acid composition, including a decrease in C18:1 and an increase in palmitic acid (C16:0) [14]. Therefore, by altering the expression of the *ACP* gene, the composition and fatty acid content of *Brassica* seed oil can be changed, which is a convenient way to improve the quality of seed oil in *Brassica* crops.

In *Arabidopsis*, the function of *ACP* genes has also been extensively reported, and these *ACP* genes have been divided into two types (plastidial *ACP* and mitochondrial *ACP*) [33,47]. In this study, we also predicted the expression of *ACP* genes in chloroplasts and mitochondria, which has been validated in tobacco. Based on our analysis of *cis*-acting elements, the *ACP* gene of *Brassica* plants plays a crucial role in light response, hormone regulation, stress resistance, and growth and development [48]. However, the number of *cis*-acting elements may vary among different species due to gene loss during differentiation and other factors [49]. These findings further support the positive role of the *ACP* gene in stress resistance. Tissue expression data and qRT-PCR analysis revealed distinct expression levels of *BnaACP* genes throughout the entire growth period of *B. napus* in all tissues. Under salt stress treatment, the expression pattern of *BnaACP* genes varied at different time points. We noticed that the most pronounced response was observed for *BnaACP4.1* and *BnaACP5.1*, which showed particular sensitivity to salt stress. After exposed to salt stress, the relative expression level of *BnaACP4.1* was significantly decreased, while that of *BnaACP5.1* showed an increase. Similar results were observed in orthologous *AtACPs.* In *Arabidopsis*, exposure to abiotic stress such as iron deficiency and nitrogen starvation led to down-regulation of *AtACP4* [14]. Knocking out *AtACP5* resulted in increased sensitivity to salt stress, while overexpressing *AtACP5* in transgenic lines exhibited improved salt tolerance compared to the wild-type [14]. Additionally, this result aligns with the predicted involvement of *cis*-acting elements in response to stress and defence. The diversity in the expression patterns of *BnaACP* genes suggests the involvement of various mechanisms in plant development and defence responses.

## 4. Materials and Methods

### 4.1. Plant Materials and Data Sources

The seeds of the tetraploid *B. napus* accession ‘Zhongshuang 11’ were preserved by our laboratory. The seeds were sterilized and germinated in Petri dishes with wet filter paper until the cotyledon was fully expanded. They were then transplanted to 1/2 Hoagland nutrient solution for a duration of 10 days. The temperature and light cycle were maintained at 22 °C/19 °C (light and darkness) with a 16 h light and 8 h darkness cycle. Subsequently, the experimental group was subjected to salt stress treatment by adding 150 mM NaCl [50,51]. Three biological replicates were set up for this purpose. Fresh leaves were collected at 1, 3, and 6 h after treatment. These leaves were immediately frozen in liquid nitrogen and stored at −80 °C for subsequent experiments.

To obtain information on the *Arabidopsis ACP* (*AtACP*) gene family, we retrieved eight protein sequences and nucleic acid sequences of the *AtACP* gene family (*AtACP1*~*AtACP5*, *mtACP1*~*mtACP3*) from the TAIR database (http://www.arabidopsis.org, accessed on 14 August 2023). The hidden Markov model (HMM) of the ACP PP-binding domain (PF00550) was downloaded from the Pfam database (http://pfam.Xfam.org/, accessed on 14 August 2023). The genome data of *B. carinata* was available from CoGe (https://genomevolution.org/coge/, accessed on 10 August 2023) under id 63922 [25], and the other five *Brassica* genomes were obtained from the BRAD database (http://brassicadB.org/brad/, accessed on 10 August 2023) [52]. The transcriptome data were downloaded from the BnIR database https://yanglab.hzau.edu.cn/, accessed on 23 August 2023) [53].

### 4.2. Identification and Characterization of ACP Genes

The *ACP* genes in allotetraploids (*B. juncea*, *B. napus*, and *B. carinata*) and their diploid progenitors (*B. rapa*, *B. nigra*, and *B. oleracea*) were comprehensively identified. In the identification process, eight ACP protein sequences from *Arabidopsis* were used as queries to perform BLASTp searches (E-value < 1 × 10^−5^) with all proteins from these six species. Furthermore, the protein sequence of the six *Brassica* species was searched using the PP-binding domain (PF00550) as a query through the hmmersearch subroutine of HMMER v3.0 [54]. The candidate genes obtained from hmmersearch and BLASTp were merged, and any repeated candidate genes were removed. These candidate genes were then verified using the SMART database (http://smart.embl-heidelberg.de/, accessed on 25 August 2023), NCBI conserved domain database (CDD, https://www.ncbi.nlm.nih.gov/cdd, accessed on 25 August 2023), and Pfam database. The physicochemical properties of the ACP gene family members in the six *Brassica* species, including MW, pI, II, and GRAVY, were analyzed using the online software ProtParam v3.0 tool (http://web.ExPASy.org/protparam/, accessed on 25 August 2023). The subcellular localization of the ACP proteins in the six *Brassica* species was predicted using the Cell-PLoc 2.0 online software [55].

### 4.3. Phylogenetic Relationship Analysis

To construct the phylogenetic tree of the ACP proteins, we aligned 120 full-length protein sequences from six *Brassica* species and eight protein sequences from *Arabidopsis* using the MUSCLE program [56]. The alignment was performed with default parameter values. The MGEA v11.0.13 [57] software was used with the ML method and the Tamura-Nei nucleotide substitution model. The stability of the phylogenetic tree was confirmed using the bootstrap method with 1000 replicates. Finally, the web service iTOL (http://itol.embl.de/, accessed on 28 August 2023) [58] was utilized to annotate the phylogenetic tree.

### 4.4. Conserved Motif and Gene Structure Analysis

The full-length ACP protein sequences of the six *Brassica* species were analyzed using the online software MEME v5.5.4 [59] to identify conserved sequences and important functional sites. Ten motifs were set, while other options were kept at default parameters. To determine the gene structure of ACP, GSDS 2.0 software (Gene Structure Display Server 2.0, https://gsds.gao-lab.org/, accessed on 29 August 2023) [60] was used to detect exon/intron composition information, following the default parameters.

### 4.5. ACP Gene Promoter Sequences and Chromosomal Localization Analysis

The promoter sequences of the *ACP* genes in the six *Brassica* species were extracted using TBtools v1.098 [61]. The *cis*-regulatory elements (CREs) in the promoter regions of the *ACP* genes were predicted using the PlantCARE server (Plant *cis*-Acting Regulatory Element, http://bioinformatics.psb.ugent.be/webtools/plantcare/html/, accessed on 29 August 2023) [62]. The chromosome localization of the *ACP* gene family members in the six *Brassica* species was mapped using the Gene Location Visualize feature of TBtools software, based on the chromosome annotation information of ACP genome files.

### 4.6. Gene Duplication, Synteny, and Evolution Analysis of ACP Genes

Gene duplication is a common occurrence in plant species and plays a significant role in the expansion of gene families. In this study, we investigated the duplication events of *ACP* genes using DupGen_finder. Subsequently, we identified interspecific and intraspecific collinear gene pairs within the *ACP* gene family across the six *Brassica* species using JCVI v1.3.5 [63]. Finally, we calculated the Ka/Ks ratio of the intraspecies and interspecies *ACP* gene family genes in these six *Brassica* species using the Simple Ka/Ks Calculator module of TBtools.

### 4.7. Analysis of Tissue Expression Characteristics and qRT–PCR

To investigate the expression levels of *BnaACP* gene family members in various tissues of *B. napus*, we downloaded transcriptome data for roots, stems, leaves, cotyledons, buds, sepals, petals, filaments, pollen, siliques, and mature seeds of *B. napus.* The expression levels of the *BnaACP* gene family members were standardized and visualized using TBtools.

Total RNA was extracted from the leaves of *B. napus* seedlings using an RNA extraction kit (Aidlab Biotech, Beijing, China). First-strand cDNA was synthesized by reverse transcription using a HiFiScript gDNA Removal cDNA Synthesis Kit. For real-time quantitative PCR analysis, six *BnaACP* genes with the highest homology to *Arabidopsis ACP* genes were selected. Specific quantitative analysis primers were designed using NCBI, with Actin (used by [64]) serving as a control (Table S6). The FQD-48 X real-time fluorescence quantitative PCR system was employed to assess gene expression levels in this study, following the method of [65]. Three biological replicates and three technical repeats per gene were prepared. The 2^−∆∆CT^ method [66] was used for the relative quantitative analysis of the gene, revealing the expression pattern of *BnaACP* genes in *B. napus* under salt stress treatment.

### 4.8. Subcellular Localization

In this experiment, the fusion protein expression method was used as follows: the GFP was fused with the C-terminal of the BnaACP protein, and the location of the BnaACP protein was determined by observing the fluorescence signal of the GFP. The specific primers (Table S6) were used to amplify the entire coding sequences (CDS) of the BnaACP. The vector was linearized using BamHI. The Hieff Clone^®^ Plus One Step Cloning Kit was utilized to subclone the CDS of six selected BnaACPs into the target vector with the GFP. The target gene, which contained the GFP fusion protein, was then introduced into tobacco leaves using Agrobacterium (GV 3101) injection. Additionally, the mitochondria-specific marker (pSuper 1300-pFAY-mCherry, Protein Interaction) was transformed using Agrobacterium (GV 3101), and the target gene with the GFP fusion protein was co-injected into the tobacco. The tobacco plants were cultured in dark conditions at 28 °C for 24 h, followed by normal cultivation for 48 h. Finally, the fluorescence signal was observed using laser confocal microscopy.

## 5. Conclusions

In this study, a total of 120 *ACP* genes with PP-binding domains were identified in the six *Brassica* species, which were mainly divided into six subclades according to the phylogenetic relationships of these *ACP* genes, and each subclade contained the same conserved motifs and domains. A comprehensive and systematic study of these genes was conducted from the aspects of gene location, gene evolution, gene structure, *cis*-acting elements, protein physical properties, gene expression patterns, and subcellular localization. Additionally, qRT-PCR was used to preliminarily explore the expression level and potential role of the *BnaACPs* gene under salt stress. By employing gene family identification and comparative genomics, this study contributes to the bioinformatics analysis of the *ACP* gene family in the six *Brassica* species, providing a theoretical foundation for future investigations on *ACP* family genes.

## Data Availability

All the data generated or analyzed during this study are included in this published article and its Supplementary Information Files. The genome data of *B. carinata* were available from CoGe (https://genomevolution.org/coge/, accessed on 10 August 2023), and the five other *Brassica* genomes were obtained from the BRAD database (http://brassicadB.org/brad/, accessed on 10 August 2023). The transcriptome data of *B. napus* were downloaded from the *B. napus* multiomics information resource database (BnIR, https://yanglab.hzau.edu.cn/, accessed on 23 August 2023). The *A. thaliana* sequences in this article were downloaded from the TAIR database (http://www.arabidopsis.org, accessed on 14 August 2023). The seeds of the tetraploid *B. napus* (cv. Zhongshuang 11) were preserved by our laboratory.

## Supplementary Materials

The following supporting information can be downloaded at: https://www.mdpi.com/article/10.3390/plants13070950/s1. Figure S1: Subcellular localization of four BnaACP proteins. GFP: green fluorescent protein. Scale bar = 20 μm. Figure S2: Conserved motifs of *ACP* genes in six *Brassica* species. Figure S3: Chromosome distribution of *ACP* genes in six cultivated species of *Brassica*. (a) *B. rapa*, (b) *B. nigra*, (c) *B. juncea*, (d) *B. oleracea*, (e) *B. carinata*. The different colours on the chromosomes represent the gene density. Figure S4: The gene expression levels of *ACP* genes in different tissues of *B. napus*. Table S1: Summary of ACP family proteins in six cultivated *Brassica* species. Table S2: Dispersed duplication events of *ACP* genes in six *Brassica* species. Table S3: Identify the interspecific and intraspecific collinear gene pairs. Table S4: The intraspecific Ka/Ks ratios analysis of duplicate gene pairs of *ACP* gene family in six *Brassica* species. Table S5: The interspecies Ka/Ks values of *ACP* families in the six *Brassica* species. Table S6: The primer sequences of *ACP* genes used for qRT-PCR and subcellular localization.
