# Correction: Qian et al. Identification, Evolutionary Dynamics, and Gene Expression Patterns of the *ACP* Gene Family in Responding to Salt Stress in *Brassica* Genus. *Plants* 2024, *13*, 950

**DOI:** 10.3390/plants14203177

**Published:** 2025-10-16

**Authors:** Fang Qian, Dan Zuo, Tuo Zeng, Lei Gu, Hongcheng Wang, Xuye Du, Bin Zhu, Jing Ou

**Affiliations:** 1 School of Life Sciences, Guizhou Normal University, Guiyang 550025, China; 82101221107@caas.cn (F.Q.); 21010100449@gznu.edu.cn (D.Z.); zengtuo@gznu.edu.cn (T.Z.); leigu1216@nwafu.edu.cn (L.G.); duxuye@gznu.edu.cn (X.D.); 201703008@gznu.edu.cn (B.Z.); 2 College of Forestry, Guizhou University, Guiyang 550025, China

In the original publication [1], there were two mistakes in Figures 1 and S1 as published. In Figure 1, the authors regret that the image of BnaACP5.1 from Figure S1 was mistakenly reused in Figure 1 due to a figure assembly error. Similarly, in Figure S1, the authors made the same unintentional mistake: the image of BnaACP5.1 and BnamtACP2.1 were inadvertently duplicated. The corrected figures appear below. The authors state that the scientific conclusions are unaffected. This correction was approved by the Academic Editor. The original publication has also been updated.

**Figure 1:**
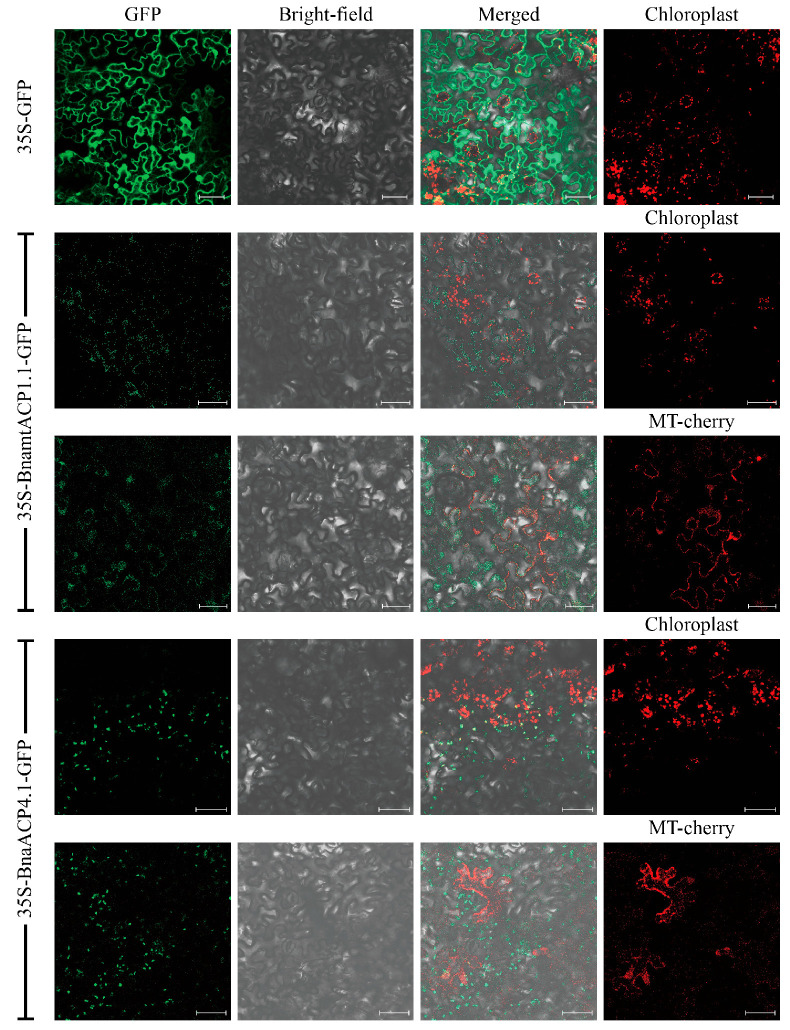
Subcellular localization of two BnaACP proteins in tobacco. Chloroplast: chloroplast auto-fluorescence; MT-mcherry: mitochondria-specific marker-labelled recombinant plasmid. Scale bar = 50 µm.

**Figure S1:**
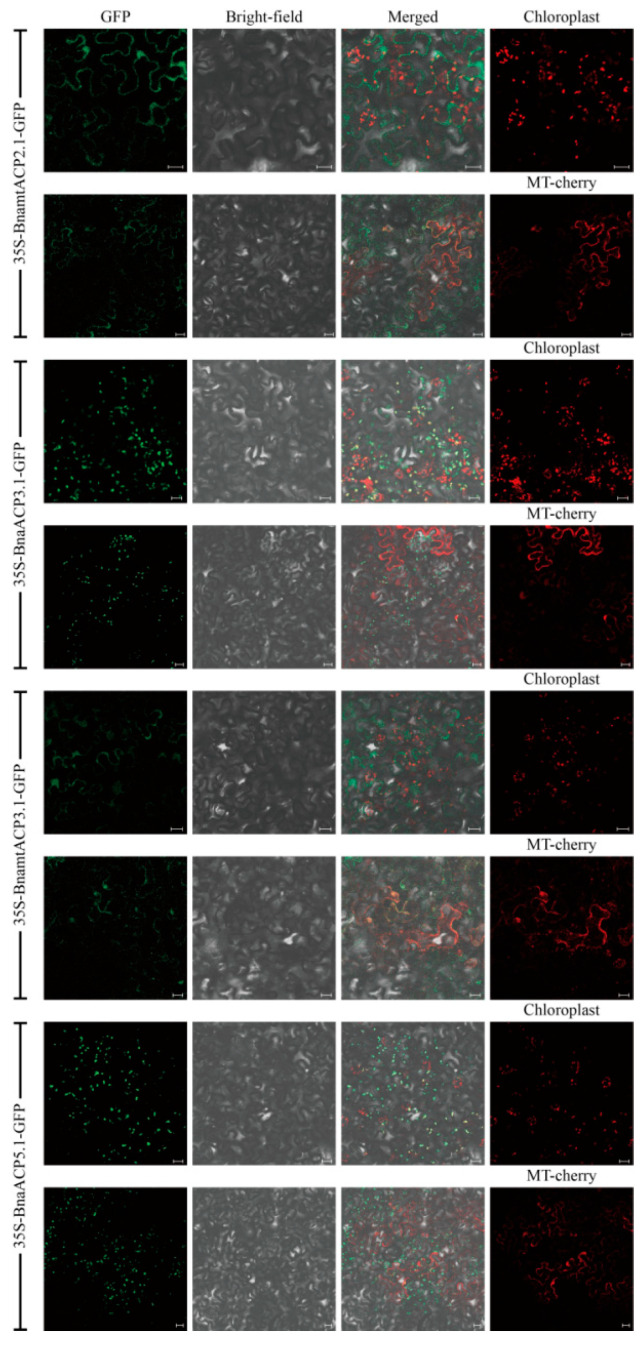
Subcellular localization of four BnaACP proteins. GFP: green fluorescent protein. Scale bar = 20 µm.
