# Supplementary material for: Identification, Evolutionary Dynamics, and Gene Expression Patterns of the ACP Gene Family in Responding to Salt Stress in Brassica Genus
Source: Plants (Basel). 2024 Mar 25;13(7):950. doi: 10.3390/plants13070950 (PMC11013218; doi:10.3390/plants13070950)
Supplement: Supplementary file 1 [file plants-13-00950-s001.zip › Supplementary Materials Figure S1-S4.pdf]

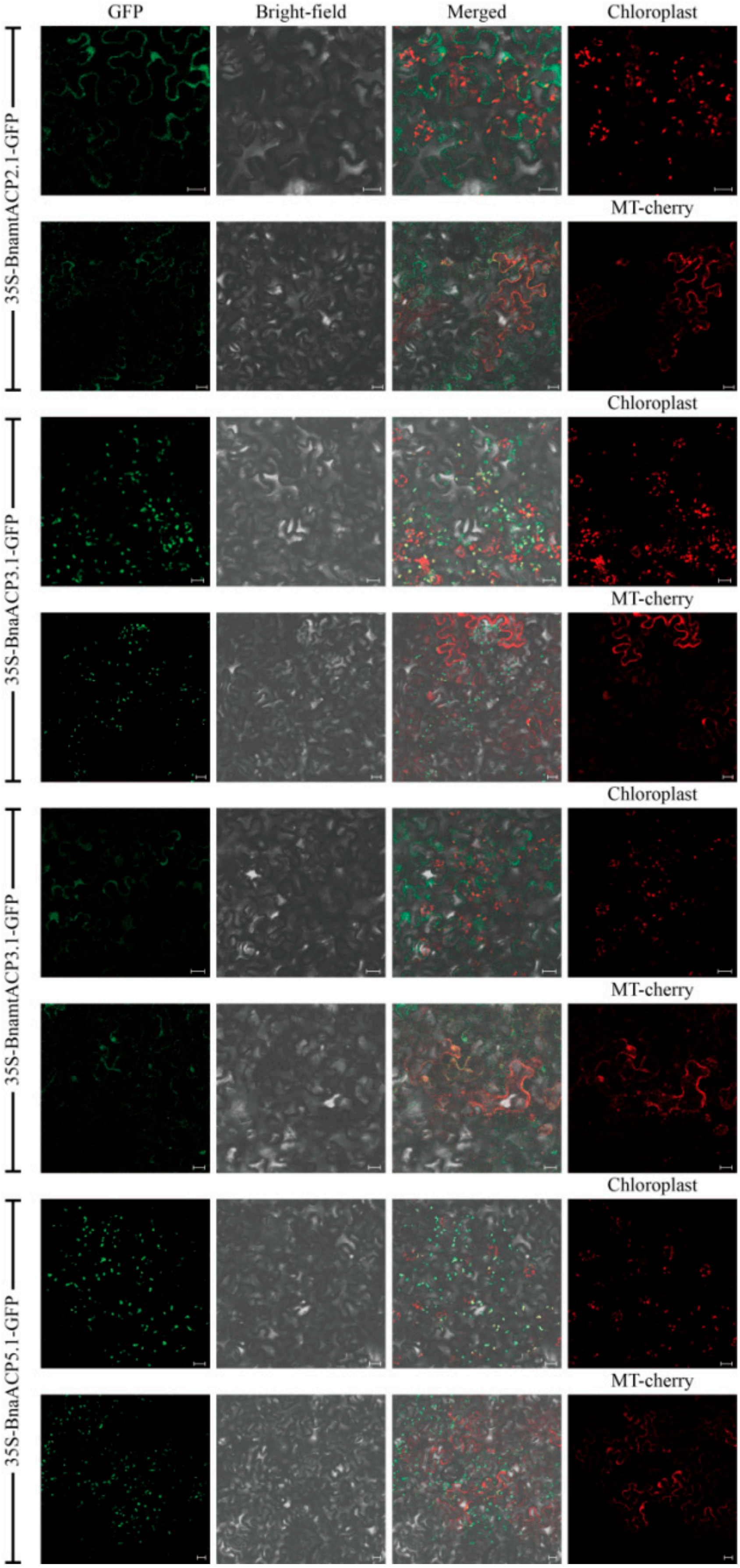

Figure S1. Subcellular localization of four BnaACP proteins. GFP: green fluorescent protein. Scale bar = 20 μm.

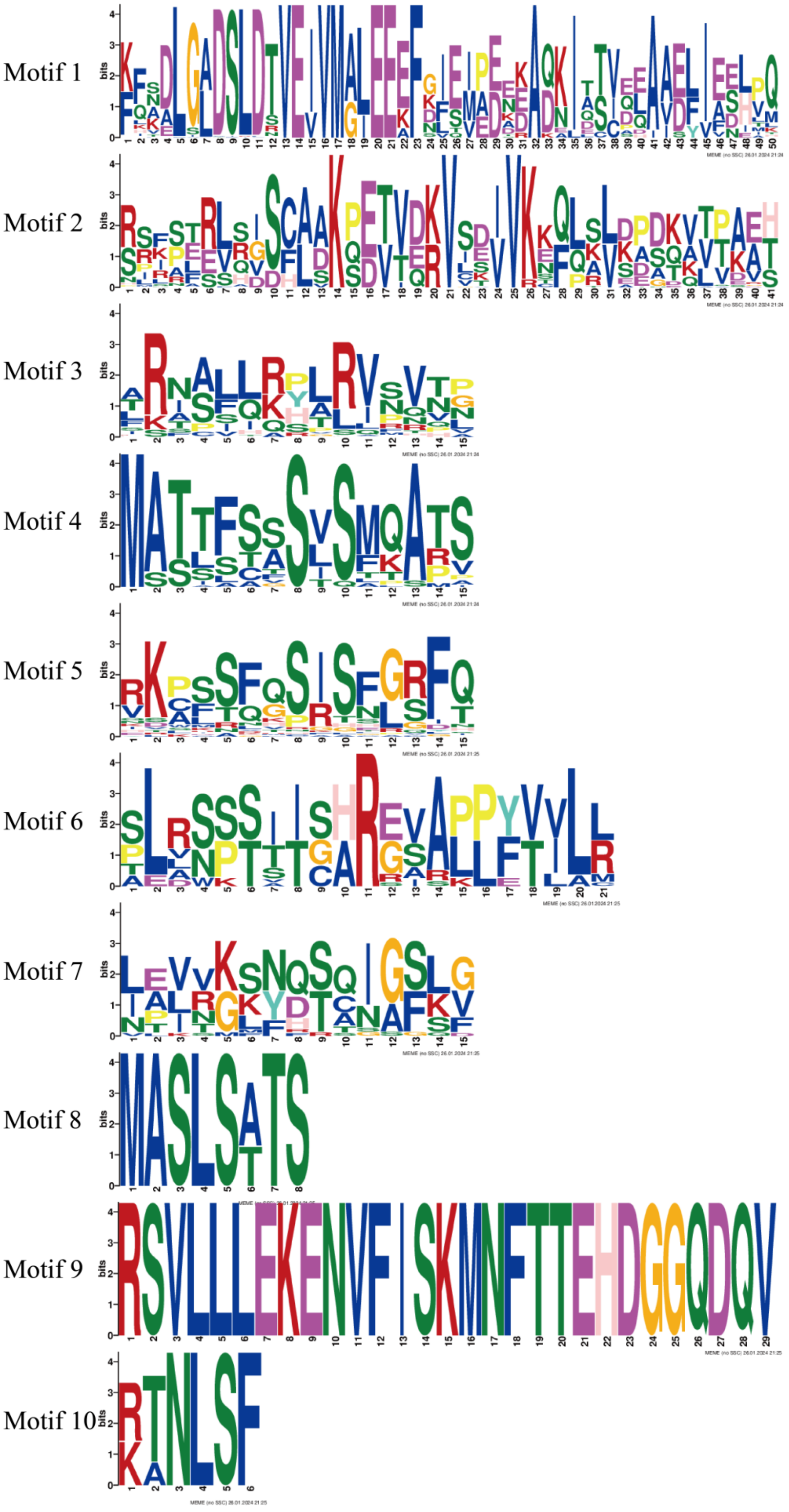

Figure S2. Conserved motifs of the *ACP* genes in six *Brassica* species.

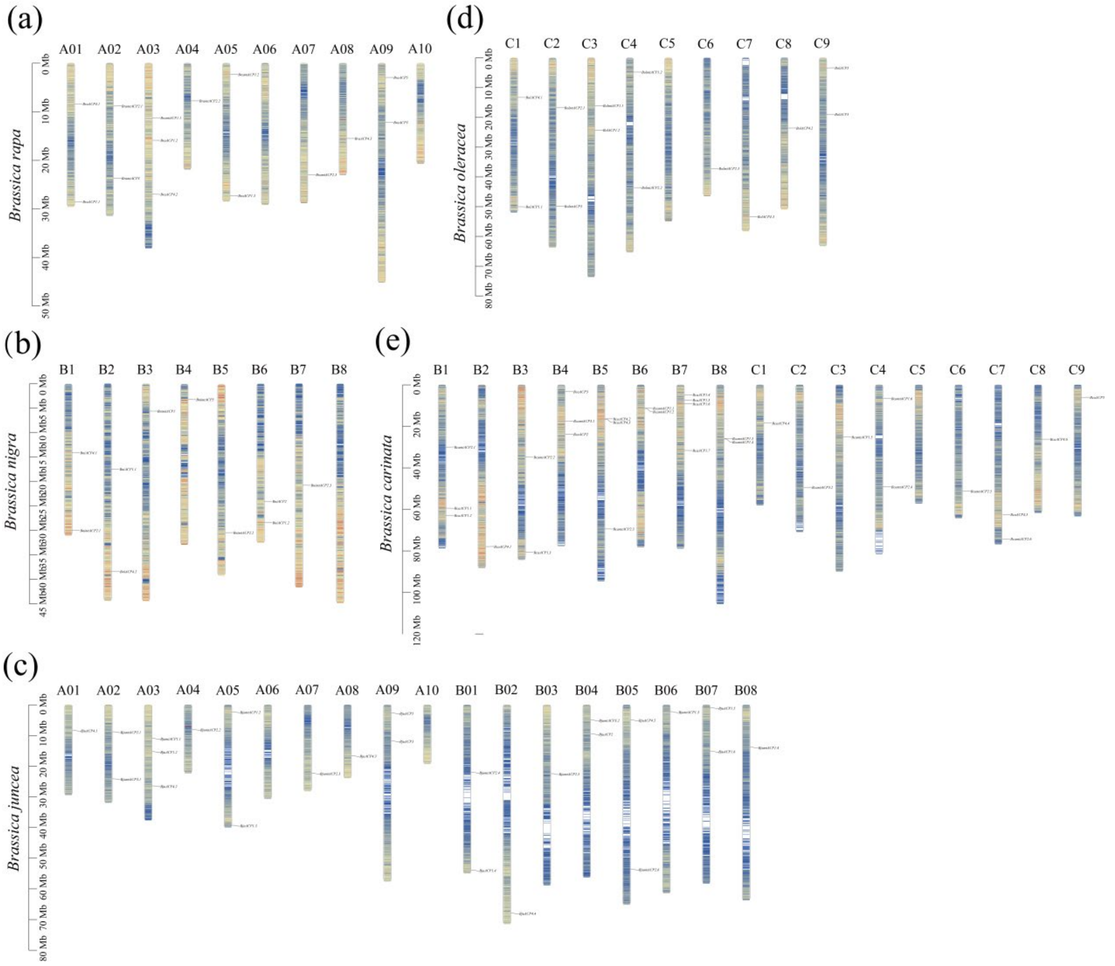

Figure S3. Chromosome distribution of *ACP* genes in six cultivated species of *Brassica*. (a) *B. rapa*, (b) *B. nigra*, (c) *B. juncea*, (d) *B. oleracea*, (e) *B. carinata*. The different colours on the chromosomes represent the gene density.

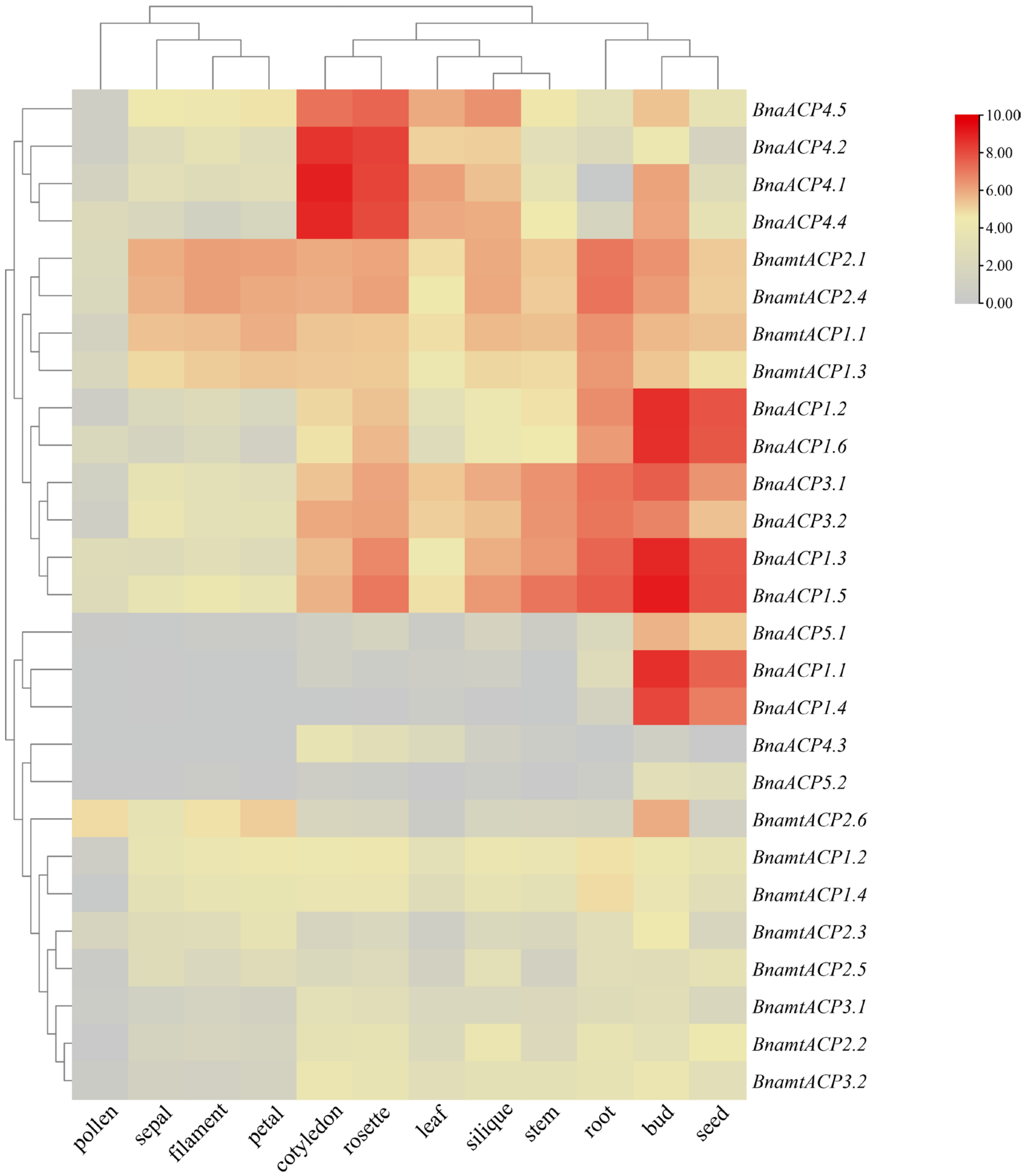

Figure S4. The gene expression levels of *ACP* genes in different tissues of *B. napus*.
